# Hippocampal Lipocalin 2: an emotional link between neurons and glia

**DOI:** 10.3389/fncel.2013.00132

**Published:** 2013-08-23

**Authors:** Oliver von Bohlen und Halbach, Hermona Soreq

**Affiliations:** ^1^Institute of Anatomy and Cell Biology, Universitätsmedizin GreifswaldGreifswald, Germany; ^2^The Edmond and Lily Safra Center of Brain Science, The Hebrew University of JerusalemJerusalem, Israel

**Keywords:** lipocalin-2, astrocytes, hippocampus, synaptic plasticity, mouse

Lipocalins are a diverse family of secreted proteins that are involved in a variety of cellular processes. A member of the lipocalin protein family is lipocalin-2. Lipocalin-2 mRNA and protein expression in the adult brain, under physiological conditions, is thought to be rather low, especially in the hippocampus (Chia et al., 2011). Moreover, lipocalin-2 seems to be expressed, at least in the adult brain, by glia cells (Chia et al., 2011; Marques et al., 2012). Lipocalin-2 has been found to be upregulated in astrocytes after neuronal injury induced e.g. by kainate (Chia et al., 2011) and it has been shown that reactive astrocytes secrete lipocalin2 to promote neuron death (Bi et al., 2013).

Based on the expression of lipocalin 2 in the brain, the data shown in the paper “Lipocalin-2 is involved in emotional behaviors and cognitive function” by Ferreira and colleagues are very interesting. The data may hint a strong interplay between glia cell function and neuronal functioning. The authors demonstrate that lack of lipocalin-2 alters behavior and cognitive functions and induces morphological changes (dendritic length and spine-densities) and that absence of lipocalin-2 impairs synaptic plasticity. These interesting findings offer the opportunity to analyze the interplay of astrocytes and neurons in more detail. There are different scenarios that may account for the effects seen in the lipocalin-2 deficient mice:

1. The observed changes may be mediated by a receptor capable of binding lipocalin, like the 24p3R (Devireddy et al., 2005), which is known to be expressed by neurons (Ip et al., 2011).
2. Gliotransmission and the tripartite synapse (Santello et al., 2012) are known to play important roles for electrophysiological properties of neurons. Deletion of lipocalin-2 may affect the population of astrocytes, leading to changes in the activity-dependent synaptic plasticity.
3. Deficiency for lipocalin-2 may have an effect upon embryonic brain development and/or modify other regulatory molecules, such as microRNAs whose other targets may be affected differently due to these changes.

In conclusion, lipocalin-2 regulation connects between cell types, behavioral paradigms and possibly developmental ones.
